# Clinical Cases

**Published:** 1885-04

**Authors:** 


### CLINICAL BUREAU.

### CLINICAL CASES.

R. B. Johnstone, M. D., Pittsford, N. Y.

June 9th, 1879.—Mrs. Bodzilke, a German, has had many children, now nursing a baby four months' old; came with her husband at about 10 A. M., *just howling with pain.* She has a sickly look, wasted frame, has an occasional short hack of a cough, pain comes and goes, lasts five minutes and absent five minutes; during pain, face very red with spots of darker red; left cheek over the diseased tooth dark purple spot; holds the head with both hands, and rolls it from side to side; don't know why she does it except the pain is so great; don't know what to do; after the pain subsides her face, with the exception of the purple spot, grows pale and ashy.

I was undecided between Coffea and Ferrum, but finally, while Coffea covered the mental state, I decided on *account of the cough, holding head in the hands and rolling it about, the purple spot on the cheek, which was permanent,* and the paroxysmal pain, to give Ferrum$^{cc}$, in water every ten minutes. They lived one and a half miles in the country, so gave one dose on the tongue dry while in the office. Her husband was five minutes in getting the horse and started for home.

June 15th, 1879.—Met Bodzilke in the post-office; asked him about his wife; said she was all right. He asked me for his bill, which he paid, remarking that he "might have saved that *four shillings*, if he had waited half an hour." I took the money and asked him why? "Vell, she no more got any pains when she got home. I haf the medicine home; you can buy him back again for twenty-five cents." I should have pulled that woman's tooth and split her jaw, or killed the nerve with kreosote and arsenic, torturing her for three or four hours, and earned my money.

More microscopes wanted to find *iron* in the two hundredth of Ferrum.

They have moved away since the above, but I learn that she is dying of consumption, and from what I can hear is still a Ferrum case. The child died the next August with a bowel trouble. I did not attend it.

December 11th, 1884.—Mrs. J., nursing a child one year old, suffers from a sharp pain in left ovary and left mamma; bloating before menstruation, with tenderness of the abdomen upon pressure. Menses delaying and scant, last too long, followed by a profuse leucorrhœa which is yellowish in color.

The pain in left mamma is of years' duration, having had it when a child. Baby refuses that breast; it is smaller than the right, more compact, and quite natural to the touch. No pain other than the infra mammary pain as above. Constipation of the bowels, movement difficult and painful, blind piles. ℞ Lycopodium $^{cc}$ in water, four doses.

December 21st, 1884.—All day has had a sharp pain in the left under jaw, decayed tooth; made several calls during the evening and noticed that the pain came on whenever going into the house, growing in intensity while in-doors. It invariably disappeared while walking from one house to the other. This evening at 11 P. M., she is almost wild with pain; *sits in a chair and cries;* wants the tooth out. The pain in abdomen has gone, also the pain in the left breast; stools are less constipated and is able to have a movement with comparatively little trouble.

℞ Pulsatilla$^{cm}$ one dose. In ten minutes the pain was all gone; the room was quite warm, and during the rest of the night slept well.

January 2d, 1885.—Menstruation came on two days late; profuse, bright red; lasted four days; was not preceded by bloating or pain; inflammatory pain gone; no more return of the toothache, although the tooth remains.

Mrs. C. H. A.—Nursing a child two months old. The baby is exceedingly cross, and requires very much attention; cries, yells, and kicks all the time. Cham., Calc., Nux-vom., etc., did no good; besides nursing, feeds the child—the feeding of which I put an end to.

December 27th, 1884.—Husband called for medicine; says his wife is almost wild with tooth situated in upper left side; in all the teeth, pain extends to the ear and to the whole side of the face; *she cannot sit still;* wanders all over the house, looks into every corner and crack, as if hunting for relief. The baby is a regular *cyclone on wheels*, twisting and squirming in all imaginable shapes, howling enough to raise the roof; sent the mother Coffea crud., three powders, 3m.; one to be taken every ten minutes, and if not better in forty-five minutes, to report again.

December 28th.—Called at the house; am informed that the pain stopped after the second dose; has one powder left; face feels numb and swollen. ℞ Puls.$^{cm}$, one powder.

December 29th.—No pain; face swollen, and an immense quantity of pus discharging through an old decayed tooth; ℞ *Sl.* At my visit on the 28th I learned that the baby continued to howl and raise Cain until he had nursed half an hour; he went to sleep; slept all night the first time since he was born; they wanted to know if the medicine put the baby to sleep; at their request left a few powders of Coffea$^{3m}$, for baby, but they have not yet needed them. The child thrown upon the nurse alone, and the mother is free from pain and happy, because the baby sleeps. Do Puls.$^{cm}$ and Coff.$^{3m}$ contain any medicine? Bring on your microscopes.

---

### E. W. Berridge, M. D., London.

(1) *Trombidium.* March 5th, 1883.—Mrs. B., for three weeks, sore pain in all abdomen, internally; *worse before stool;* during stool, sharp pains darting downward in right abdomen. For two days, urgent, loose stool on rising from bed, and twice afterward. One dose of *Trombidium*$^{200}$ was given; the patient improved the same day, and soon recovered.

(2) *Medorrhinum.*—One dose of 10m (Fincke) removed a cough on lying down, but relieved by lying on stomach. This verifies Swan's provings with this nosode, which will be published as soon as the complete records of all the provers are obtained.

(3) *Belladonna.*—A domestic servant at twenty-four had had for three days pain in left face up to temple; worse at night as soon as she gets warm in bed, preventing sleep; worse by lying on right side; the pain is like strings drawn up toward temple and twisted round in a knot; *it comes and goes suddenly.* Has taken *Bry.* without result. I gave her a dose of *Bell.*$^{cm}$ (F. C.) at once, and ordered her to repeat it every two hours till better. The pain ceased in a few minutes after the first dose, but she finished the medicine; the pain did not return.

---

### Edward Rushmore, M. D., Plainfield, N. J.

Case I.—Intermittent fever. Miss B., when visited on the 28th of last June, had felt ill for several days, and complained then of wandering pains in the joints, worse from motion; the flesh sore to touch; pain in region of spleen; eruption like nettlerash on the legs below the knees; she felt chilly this morning, and is now hot. She received China$^{900}$, one dose. When visited the next forenoon she said she had been attacked with perspiration at 1 A. M., which lasted till 7 A. M., when she had a decided chill, followed with light heat, but with no sweat. This irregular and unusual order of symptoms did not suggest any remedy, and it was concluded to give no medicine and await the progress of the case under the former and apparently well indicated remedy. In the afternoon I was called to see her again. At 11 A. M. she had had a shaking chill with pains in the wrists, knees, and ankles, followed with heat without pains, then, sweat on the back, head, face, and hollow of the elbows. Here appeared to be a clear case, so far as known to the prescriber. The only remedy having all these concomitants of chill is Podophyllum. She received at 5 P. M. one dose in the 1,000th potency. The next morning at 9 o'clock she had a chill, with thirst for hot drink, but without the pains of the previous day. She got no more medicine and had no more chill, except once slightly, three weeks later, just after sea-bathing, and is in much improved health. I have several times prescribed Podophyllum in intermittent fevers guided by these concomitants, sometimes strengthened by loquacity in the early stages and sleepiness with the sweat, and it has not yet failed to cure.

CASE II.—Chronic catarrh. April 3d, 1884. Mrs. C. has had catarrh for five or six years; the mucus seems to run from the head to the throat. She can hawk it out rather easily. She describes the mucus as white, thick, and tasteless, worse in the early part of the day. The breath is offensive, worse in the evening. The catarrh alternates with leucorrhœa since she passed the climaxis two years ago. The leucorrhœa is attended with backache and is generally worse in the morning. She feels very weak, her food seems not to nourish her. She has to swallow often. Kali carb.$^{cm}$ (Skinner), one dose, dry, on the tongue.

April 14th.—She says something has acted like a charm. The catarrh is much less, the throat much clearer, the swallowing less frequent. Has better appetite and more relish for food. Has a feeling of weakness in the back and lower abdomen. No medicine.

April 28th.—Has gained; is stronger. Has a slight return of the catarrh, but the leucorrhœa is better. No medicine.

July 5th.—No more catarrh. She has not felt as well for many years as she does now.

Several months later I again inquired of her, and she said there had been no return of the symptoms.

